# Enabling infinite *Q* factors in absorbing optical systems

**DOI:** 10.1515/nanoph-2023-0281

**Published:** 2023-08-01

**Authors:** Radoslaw Kolkowski, Andriy Shevchenko

**Affiliations:** Department of Applied Physics, Aalto University, P.O.Box 13500, Aalto FI 00076, Espoo, Finland

**Keywords:** bound state in the continuum, optical absorption, resonant metasurface

## Abstract

Resonant optical structures have widespread applications in science and technology. However, their quality (*Q*) factors can be significantly deteriorated, if some of their parts exhibit optical absorption. Here, we show that by coupling a lossy mode of such a structure to two independent lossless modes, one can create a nonradiating and absorption-free bound state in the continuum (BIC). The *Q* factor of such a BIC is theoretically unlimited despite interaction with an absorbing structure. We use this mechanism to design a plasmonic metasurface with *Q* factors that are close to 10^7^ in the visible spectral range. The proposed mechanism is general and can be used to engineer ultrahigh-*Q* resonances in various systems containing absorbing structures.

## 1 Introduction

Resonant optical systems are an essential part of modern science and technology, enabling the operation of lasers and providing the means for controlling various light–matter interactions. The main parameter describing such systems is the *Q* factor, which quantifies the average amount of time each photon spends trapped inside the resonator before it either escapes or gets absorbed. Recently, the concept of bound states in the continuum (BICs) has provided an efficient strategy for eliminating the radiation loss [1, 2], making it possible to realize high-*Q* resonances in a diverse variety of non-absorbing optical systems [3, 4], including photonic crystals [5, 6], metasurfaces [7–10], and individual dielectric nanoresonators [11, 12]. The BIC concept originates from quantum mechanics and is introduced for ideal states with infinite *Q* factors. In theory, the outgoing radiation produced by such states is completely cancelled. As a result, the states are fully decoupled from the surrounding medium and oscillate without decay. However, experimentally achievable optical states based on this concept are never perfect and necessarily exhibit a finite optical loss, e.g., due to leakage of light associated with finite size of the system and scattering by its fabrication imperfections. The highest *Q* factors achieved in non-absorbing photonic structures using the BIC concept are on the order of 10^6^ [13, 14], which is not far from the state-of-the-art photonic crystal cavities that can achieve *Q* factors on the order of 10^7^ [15, 16]. Therefore, optical states based on the BIC concept (for brevity, we refer to them as BICs throughout the further text) have been considered promising for a range of applications [17–20], e.g., in laser technology [21–24] and nonlinear optics [25–28].

Apart from the BICs, the mechanism of destructive far-field interference has been used to enhance light confinement in other systems, such as anapoles [29, 30], trapped modes [31–35], and dark (subradiant) surface lattice resonances (SLRs) [36–38]. In the case of anapoles, the destructive interference occurs between radiation patterns of local currents that oscillate as different multipolar modes. In other cases, the suppression of outgoing radiation is due to Fano-like interaction between a broadband and a narrowband mode. These modes can be either the localized modes of individual building blocks of the system (trapped modes) or the localized resonances and Rayleigh anomalies in optical diffraction at periodic structures (SLRs). The suppression of radiative loss due to interference of various radiation channels can lead to spectrally localized reduction of absorption by preventing the resonances from being excited. The effect is similar to the electromagnetically induced transparency (EIT) [39–42]. In contrast, BICs are the eigenmodes that are inherently decoupled from any external radiation regardless of their excitation. However, if the cancellation of the outgoing radiation is determined by the symmetry and/or topology of the system and its resonant states, the phenomena referred to as trapped modes or dark SLRs are essentially equivalent to BICs. For example, in Ref. [43], it has been shown that diffractive metasurfaces made of periodically arranged magnetic and electric dipole scatterers can support trapped modes that correspond to the symmetry-protected BICs. From the practical point of view, suppression of radiative damping has been a highly successful strategy, e.g., leading to SLRs with *Q* factors not achievable with isolated plasmonic nanoparticles [44, 45]. They have been explored for plasmonic distributed feedback lasing [46–48] and strong light–matter coupling [49, 50]. Subradiant SLR band edges are especially interesting for such applications, acting as high-*Q* resonant cavities with strongly enhanced density of optical states. The BIC concept is increasingly more often used to engineer such systems [51–53].

Despite recent developments in the area of plasmonic BICs, structures made of absorbing materials have always been regarded as detrimental to the *Q* factors and not suitable for ultrahigh-*Q* systems [54], unless absorption can be compensated for by gain [55]. This is because destructive interference in the far-field reduces the radiation loss of optical modes, but their absorption loss remains unaffected, if not increased [56–59]. On the other hand, absorption can be suppressed via destructive interference in the near-field [60–62], which typically occurs under different conditions than those required by BICs. This has been the reason for relatively modest *Q* factors, on the order of 10^2^ – 10^3^, achieved by BICs in plasmonic and hybrid plasmonic-dielectric structures [63–72]. Consequently, absorbing materials, such as metals and some high-index semiconductors have been avoided when realizing BICs and other types of ultrahigh-*Q* resonators, despite the fact that these materials can be very efficient in controlling optical fields [73–75].

In this work, we discover a simple and general mechanism leading to a BIC in which *both* the radiation and absorption losses are eliminated. Such a BIC can be realized by coupling the lossy oscillator to two initially lossless optical modes. The losses due to both the radiation and absorption are *simultaneously suppressed* when the two lossless modes have equal resonance frequencies. The loss cancellation is completely determined by the eigenmode structure of the system and does not rely on avoiding the excitation, as in the case of trapped modes [32] and other EIT analogs [40–42]. Furthermore, there are no explicit symmetry requirements for the coupled modes as long as the coupling mechanism is preserved. This makes the presented BIC very general compared to other types of BICs that are subject to various symmetry constraints. Destructive interference of the coupled modes makes the underlying mechanism similar to the previously studied Friedrich–Wintgen BICs [76] that have been observed in systems with avoided crossings [64, 67–69, 77, 78]. Some of these systems exhibited a reduction of the absorption loss [78]. However, the possibility to completely eliminate the absorption loss together with the radiation loss has not been in the focus of previous works.

Using numerical simulations, we demonstrate the existence of such BICs in a realistic physical system – a periodic metasurface composed of a slab waveguide and an array of metal nanoparticles [79, 80]. By this example, we show that the proposed BICs can originate from modes of different types, such as TE and TM guided modes. In this case, the coupling results in a BIC with a mixed TE-TM polarization state. The hybrid resonances we introduce here can be perfectly lossless, even though the coupling of the modes is provided by lossy nanostructures. Our findings suggest that even strongly absorbing plasmonic or semiconductor materials can be used to construct ultrahigh-*Q* resonant systems based on BICs. The unique properties of such materials, combined with those of BICs, can lead to superior photonic devices with new functionalities.

## 2 Mechanism of loss cancellation

Consider a system of coupled harmonic oscillators described by the following equations:

(1) $$\frac{{\text{d}}^{2}x_{p}}{dt^{2}}+\gamma_{p}\frac{dx_{p}}{dt}+\omega_{p}^{2}x_{p}-\underset{q\neq p}{\sum }\Omega_{pq}^{2}x_{q}=0,$$

where *x*_*p*_ is the instantaneous amplitude of a *p*^th^ oscillator, *γ*_*p*_ the damping rate, *ω*_*p*_ the resonance frequency, and Ω_*pq*_ the coupling rate between the oscillators *p* and *q*. Assuming the ansatz *x*_*p*_ = *x*_*p*,0_e^−i*ωt*^ and using the approximation |*ω*_*p*_ − *ω*| ≪ *ω*, the above set of equations can be simplified into a linear eigenvalue problem [81]: det(***H*** − *ω* ***I***) = 0. Here, ***I*** is the identity matrix, whereas the Hamiltonian ***H*** has the diagonal terms $\omega_{p}-\frac{i}{2}\gamma_{p}$ and off-diagonal terms $\kappa_{pq}=\frac{1}{2}\Omega_{pq}^{2}/\bar{\omega }$ with $\bar{\omega }\approx \left(\omega +\omega_{p}\right)/2$.

**Figure 1:**
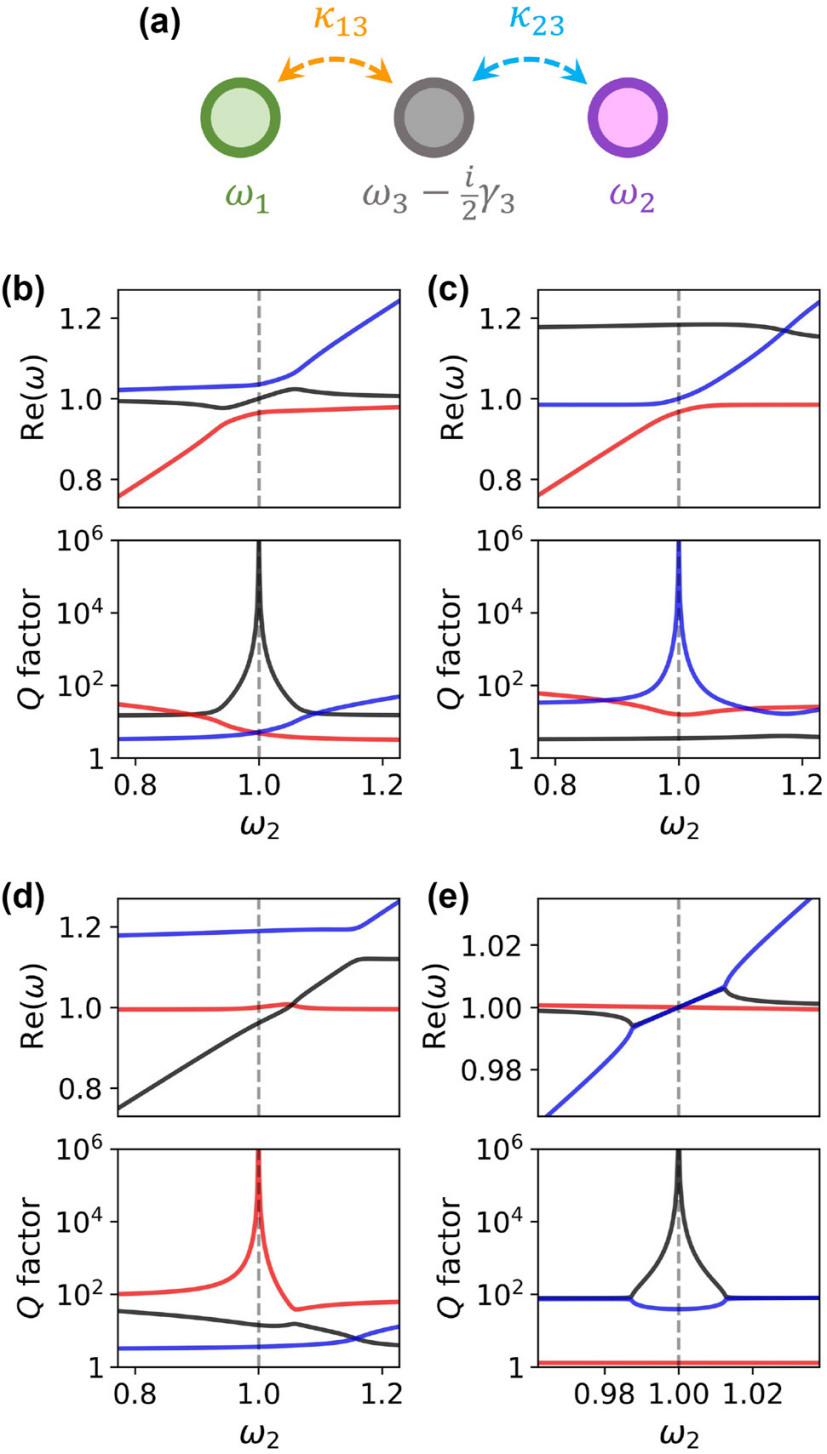
Loss cancellation. (a) Illustration of the coupling between three oscillators described by Hamiltonian ***H*** in Eq. (2). The plots in (b)–(e) show the real parts of the complex eigenvalues *ω* (top) and the corresponding *Q* factors ($Q=\frac{1}{2}Re\left(\omega \right)/Im\left(\omega \right)$, bottom) of the eigenstates of Hamiltonian ***H***, as functions of the resonance frequency *ω*_2_. The plots in (b) show the resonant case (*ω*_3_ = *ω*_1_) in which the BIC coincides with the lossy resonance, whereas (c) and (d) correspond to the off-resonant scenario (*ω*_3_ = 1.15*ω*_1_). The plots in (e) correspond to a strongly non-Hermitian system (with *ω*_3_ = *ω*_1_) in which the BIC is accompanied by a pair of exceptional points instead of an avoided crossing. In all plots, we assume *ω*_1_ = 1, whereas the other parameters are: *κ*_13_ = *κ*_23_ = 0.075 and *γ*_3_ = 0.4 in (b) and (c), *κ*_13_ = 0.045, *κ*_23_ = 0.105, and *γ*_3_ = 0.4 in (d), and *κ*_13_ = *κ*_23_ = 0.05 and *γ*_3_ = 0.8 in (e). Different colors (red, blue, black) correspond to the different eigenmodes of the system.

Now, consider a system composed of two oscillators with resonance frequencies *ω*_1_ and *ω*_2_ that are not directly coupled to each other (*κ*_12_ = 0) and show no losses (*γ*_1_ = *γ*_2_ = 0). Next, we introduce a third oscillator with resonance frequency *ω*_3_ and damping rate *γ*_3_ coupled to the first and second oscillators via *κ*_13_ and *κ*_23_, respectively (see Figure 1(a)). This system of oscillators is described by the Hamiltonian

(2) $$H=\left(\begin{array}{ccc} \omega_{1} & 0 & -\kappa_{13}\\ 0 & \omega_{2} & -\kappa_{23}\\ -\kappa_{13} & -\kappa_{23} & \omega_{3}-\frac{i}{2}\gamma_{3} \end{array}\right).$$

In the context of optical resonances, the eigenstates of this Hamiltonian are quasi-normal modes [82–84] associated with complex eigenvalues *ω*, where Im(*ω*) describes the losses. A somewhat similar three-mode system has been considered as a generalization to the Friedrich–Wintgen BIC [68, 85] and as a classical analog of the double electromagnetically induced transparency [86]. Solving the eigenvalue problem yields the following relation:

(3) $$\begin{array}{ll} & \left(\omega_{1}-\omega \right)\left(\omega_{2}-\omega \right)\left(\omega_{3}-\frac{i}{2}\gamma_{3}-\omega \right)\\ & \;=\kappa_{23}^{2}\left(\omega_{1}-\omega \right)+\kappa_{13}^{2}\left(\omega_{2}-\omega \right). \end{array}$$

For *ω*_1_ = *ω*_2_, this equation can be written as

(4) $${\left(\tilde{\omega }-\omega \right)}^{2}\left(\omega_{3}-\frac{i}{2}\gamma_{3}-\omega \right)=\left(\kappa_{13}^{2}+\kappa_{23}^{2}\right)\left(\tilde{\omega }-\omega \right),$$

where $\tilde{\omega }=\omega_{1}=\omega_{2}$. The above relation clearly shows that the system under consideration hosts a lossless hybrid eigenstate at $\omega =\tilde{\omega }$ that is fully independent of the elements *κ*_13_, *κ*_23_, and $\omega_{3}-\frac{i}{2}\gamma_{3}$. Most importantly, this eigenstate is completely immune to losses expressed by *γ*_3_, which may include also the absorption loss. This is in contrast to the Friedrich–Wintgen BIC emerging from two resonances with radiation losses only [1, 76].

The appearance of a lossless BIC in the above system is illustrated in Figure 1(b)–(e), where the eigenvalues of ***H*** are plotted as functions of the resonance frequency *ω*_2_. The BIC is present at *ω*_2_ = *ω*_1_, regardless of the choice of parameters *ω*_3_, *γ*_3_, *κ*_13_, and *κ*_23_. In particular, Figure 1(b) shows the resonant scenario (*ω*_1_ = *ω*_3_), whereas in Figure 1(c), the lossy resonance of frequency *ω*_3_ is detuned from *ω*_1_ by approximately the half-width of the resonance peak. The BIC would also emerge if the rest of the parameters were chosen differently, giving rise to hybrid modes of a qualitatively distinct character. For example, if *κ*_13_ ≠ *κ*_23_ or *κ*_13/23_ ≪ *γ*_3_, one can obtain the accidental degeneracies and exceptional points [69, 87] in addition to the BIC, which is illustrated in Figure 1(d) and (e).

A relatively similar mechanism to eliminate absorption is that of the electromagnetically induced transparency [39]. In our case, however, the loss cancellation occurs by interference of three excited modes instead of two driving fields, and furthermore, it is entirely determined by the eigenmode structure of the system. This makes the proposed mechanism different from optical systems analogous to EIT [32, 40–42]. In such systems, absorption can be eliminated by rendering the resonant modes completely dark, which prevents their excitation. Such situation occurs, for example, in metamaterials, in which Fano-like interference gives rise to trapped modes and a transparency window in the excitation spectrum [32]. In such a case, however, the trapped modes can be “absorption-free” only if their excitation is avoided or if the structure is made of non-absorbing materials. In contrast, our hybrid mode would remain perfectly lossless even if it were excited internally, e.g., by a source embedded in the structure. From this point of view, our states are similar to the symmetry-protected BICs [7, 88, 89] and Friedrich–Wintgen BICs [64, 67–69, 77, 78], in which the modes can exhibit a reduced absorption loss due to their local interference in the near-field, that can be destructive to some degree [68, 78]. However, the coupling mechanism presented here ensures that simultaneous cancellation of the radiation and absorption losses depends only on the frequency matching of the modes and takes place without any specific symmetry requirements, as long as the coupling mechanism is retained.

The proposed mechanism can be implemented in various physical systems. One of them is a linear array of coupled waveguides. In a configuration of three parallel waveguides, the middle one can be lossy, while the other two lossless. If the two side waveguides are coupled only through the central waveguide, then the propagation of light in the array can be described by the same Hamiltonian as in Eq. (2). The main difference between the coupled waveguides and coupled oscillators is that, in the case of coupled waveguides, the eigenvalues *ω* should be interpreted as the propagation constants of the eigenmodes, and not as their eigenfrequencies. One eigenmode, in which absorption is eliminated, has an antisymmetric field distribution with zero amplitude in the middle waveguide and out-of-phase oscillation in the side waveguides [90, 91]. This eigenmode would propagate without losses despite the fact that the two waveguides on the sides are coupled through the lossy waveguide.

A slightly more complex optical system that realizes the proposed mechanism would be a system of three coupled ring resonators, in which the central resonator is lossy. One may expect an eigenmode, in which light is trapped in the two lossless resonators, avoiding the lossy one. However, the coupling would still occur through the lossy resonator, locking the relative phase of the fields in the lossless resonators. The fields of the two coupled modes in the lossy resonator will disappear due to destructive interference. Other types of optical resonators can also be considered, e.g., Fabry–Pérot cavities [92], photonic crystal cavities [93], antenna-cavity hybrids [94–96], optomechanical resonators [97], and elementary quantum oscillators, such as atoms and molecules [49]. The presented BIC would also have analogs in physical systems beyond optics, e.g., in purely mechanical oscillators (starting with the classic example of coupled pendulums), acoustic resonators [98], and electric circuits [99].

## 3 A plasmonic metasurface

**Figure 2:**
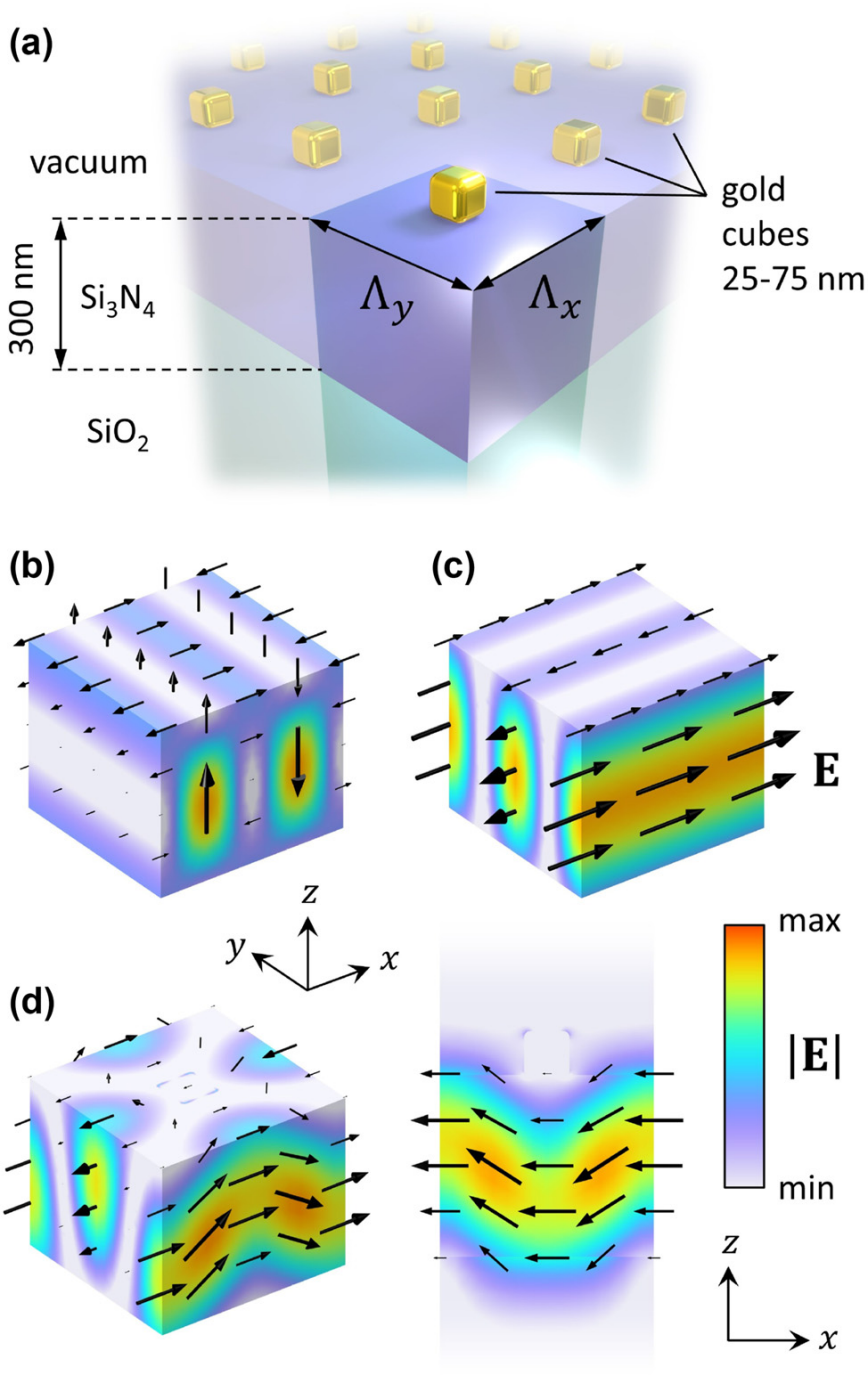
BIC in a plasmonic metasurface. (a) Schematic illustration of a metasurface supporting hybrid BICs. The unit cell and the two lattice periods Λ_*x*_ and Λ_*y*_ are highlighted in the foreground. In (b), (c), and (d), the spatial distributions of the electric field norm (color) and instantaneous electric field vector (black arrows) are shown. They were obtained using COMSOL Multiphysics. In (b) and (c), two eigenmodes of a bare Si_3_N_4_ waveguide at the Γ point are presented: (b) TM mode, forming a standing wave along *x* with a node in the center of the unit cell; (c) TE mode, forming a standing wave along *y* with an antinode in the center of the unit cell. (d) A hybrid quasi-BIC excited in a metasurface by a plane wave polarized along *x* and normally incident from the top (*λ* ≈ 645.43 nm, Λ_*x*_ = 352.5 nm, Λ_*y*_ = 340 nm, nanocube size 75 nm): 3D view of the unit cell (on the left) and cross-cut in the *xz*-plane (on the right).

To demonstrate the capabilities of the proposed BIC, we consider a periodic metasurface composed of a 2D array of metal nanoparticles on the surface of a lossless slab waveguide (see Figure 2(a)). We assume that the metasurface is infinite in the transverse directions, such that it can be described using periodic boundary conditions with the in-plane momentum **k**_‖_. Hybrid optical metasurfaces of this type have been studied previously in the context of plasmonic-photonic resonances known as waveguide-plasmon polaritons (WPPs) [79, 80]. In this work, we investigate the coupling between the TE and TM modes mediated by an array of lossy nanoparticles, giving rise to a hybrid BIC with suppressed absorption loss rate and a mixed TE-TM polarization state. Obviously, there can be found other coupling configurations for this demonstration, e.g., coupling between TE modes in a square lattice, or coupling between two counter-propagating modes in a simple one-dimensional grating. However, the purpose of the example we have chosen is to show that the proposed BIC can be formed independently of any specific symmetry requirement. The two modes coupled by metal nanoparticles in our example have different polarizations and different field profiles. Moreover, the structure under consideration lacks the up-down mirror symmetry that is required by the Friedrich–Wintgen BICs [77, 100].

In the numerical calculations, the metal nanoparticles are gold nanocubes with the rib size ranging from 25 to 75 nm on the surface of a 300 nm thick waveguide with a Si_3_N_4_ core and a SiO_2_ substrate. The optical constants of Si_3_N_4_, SiO_2_, and Au are taken from Refs. [101–103], respectively. The refractive index of the substrate (1.46) is considerably lower than that of the core (2.04), which provides efficient confinement for light guided in the core for the chosen thickness. In fact, the nanoparticle size, shape, and material composition are not critical for the emergence of the proposed BIC and can be chosen arbitrarily. However, we intentionally select nanoparticles that exhibit an electric-dipole-like localized surface plasmon resonance (LSPR) in the visible spectral range, which makes them strongly polarizable by the in-plane polarized evanescent fields of the guided modes. We also deliberately choose gold instead of silver as the nanoparticle material, as it shows significant optical absorption in the visible spectral range. This allows us to demonstrate the efficiency of our approach. In addition, the metasurface design is to some extent driven by the feasibility of its experimental realization and its possible future applications in spectroscopy. For example, a thin waveguide typically requires a solid substrate as a mechanical support, but a free-standing metasurface would be equally sufficient for the purpose of the numerical calculations presented here.

Let us first consider a slab waveguide with a periodic perturbation of infinitesimal strength, i.e., a virtually periodic waveguide in the absence of the nanoparticles. Such a waveguide supports mutually orthogonal TE and TM guided modes described by the effective mode indices *n*_TE_ and *n*_TM_. Due to the periodicity of the system, each of the guided modes forms an onset of standing waves at the Γ point (|**k**_‖_| = 0). These standing waves correspond to the band edges of the diffractive resonances with resonance frequencies *ω* governed by the 2^nd^ Bragg condition (*ω* = 2*πc*/*n*Λ for the mode index *n* and lattice period Λ). The resonance frequencies for the TE and TM modes can be matched by tuning the periods along the two lattice directions (*x* and *y*), e.g., such that *n*_TE_Λ_*y*_ = *n*_TM_Λ_*x*_. We investigate these modes using the eigenfrequency analysis (in the absence of an incident field) in COMSOL Multiphysics. By looking at the electric field distributions in Figure 2(b) and (c), one can clearly see that the TM and TE standing waves formed along the *x* and *y* axes, respectively, are both polarized parallel to the *x*-axis at the center of the unit cell on the waveguide surface. Hence, both these modes can resonantly couple to each other through surface-mounted nanoparticles.

The hybridization of the TE and TM standing waves mediated by the nanoparticles gives rise to three new resonances, including a hybrid dark resonance shown in Figure 2(d). This resonance is excited by a normally incident plane wave polarized along *x* and propagating in the negative *z*-direction, and the presented results were obtained by a scattering simulation in COMSOL. The same excitation conditions are assumed in all other calculations presented further in this article. The incident polarization is chosen to match the orientation of the electric field vector of the TE and TM modes at the positions of the nanoparticles. Although in total, there are eight TE and TM standing waves at the Γ point, the contribution of the other modes can be neglected. Hence, the description of the hybrid metasurface under consideration can be based on the Hamiltonian ***H*** of Eq. (2). The TE and TM standing waves can also individually hybridize with the nanoparticles when the system is tuned away from the TE-TM matching condition. In such a case, the hybridization gives rise to simple WPPs.

**Figure 3:**
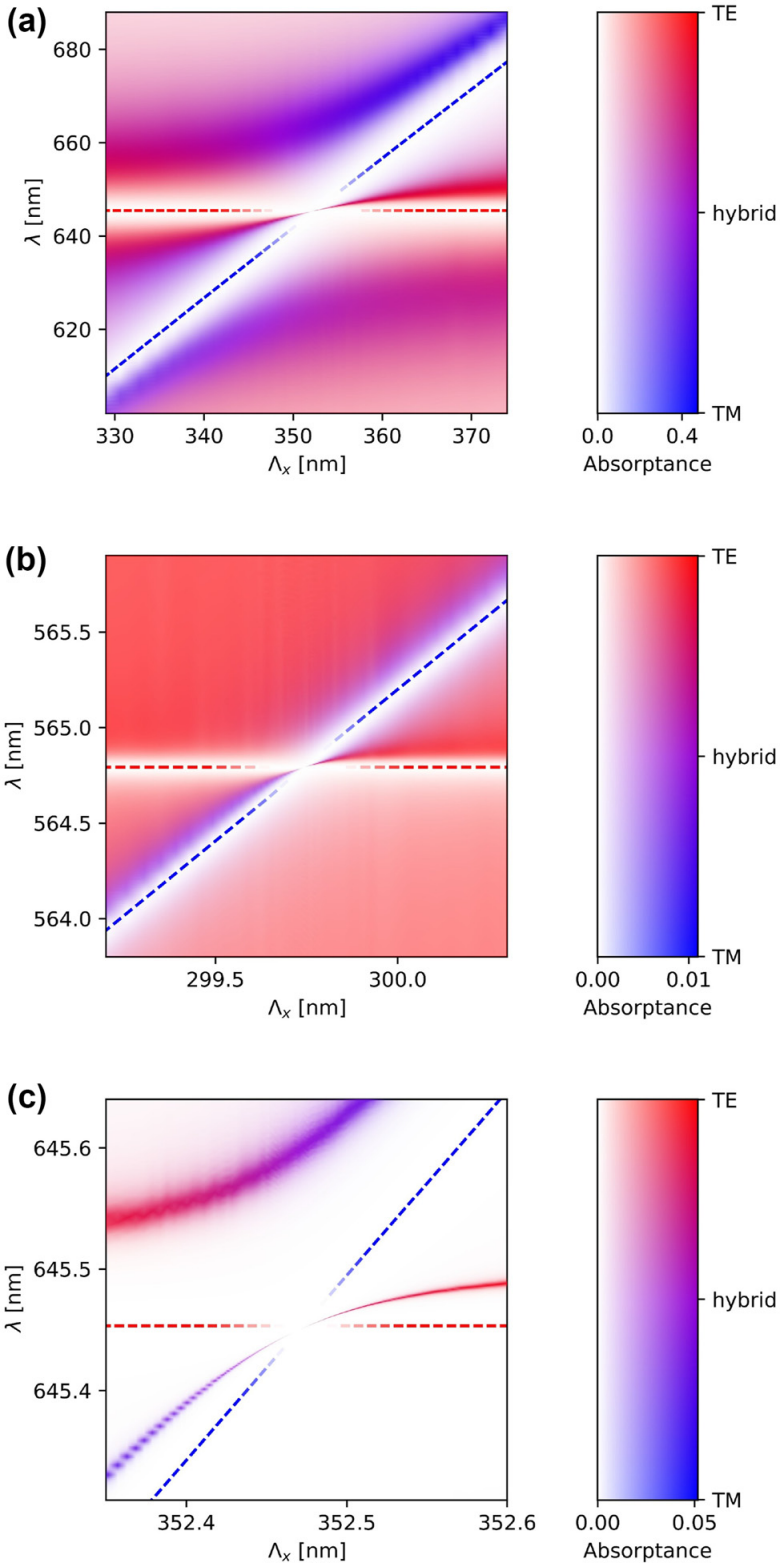
Hybrid quasi-BICs revealed in metasurfaces with various geometrical parameters. The absorptance and the average polarization state of the excited electric field are plotted as functions of period Λ_*x*_ (horizontal axis) and incident wavelength *λ* (vertical axis). The absorptance is encoded in the opacity of the data points (displayed on a white background), whereas the polarization is encoded in the colors: red for the pure TE polarization, blue for the pure TM polarization, and violet for the hybrid polarization. The red and blue dashed lines illustrate the Bragg condition for the TE and TM guided modes, respectively. These lines are made transparent near the quasi-BICs not to obscure them. The graph in (a) corresponds to metasurfaces with nanocubes of size 75 nm, whereas in the remaining graphs, the size of nanocubes is set to 25 nm. In (b), Λ_*y*_ = 292 nm, whereas in (a) and (c), Λ_*y*_ = 340 nm. In (a) and (b), the quasi-BIC overlaps with the LSPR (a resonant scenario), whereas in (c), the quasi-BIC is away from the LSPR (an off-resonant scenario). The values of absorptance and polarization state were obtained using COMSOL Multiphysics.

Figure 3 shows the optical properties of the hybrid metasurfaces at normal incidence as functions of period Λ_*x*_ and wavelength *λ*. The color represents the average polarization state of the electric field excited in the waveguide. This average polarization state is calculated as $\left(\left\langle I_{\text{TE}}\right\rangle -\left\langle I_{\text{TM}}\right\rangle \right)/\left(\left\langle I_{\text{TE}}\right\rangle +\left\langle I_{\text{TM}}\right\rangle \right)$, where $\left\langle I_{\text{TE}}\right\rangle =\left\langle |E_{x}{|}^{2}+|E_{y}{|}^{2}\right\rangle$ and $\left\langle I_{\text{TM}}\right\rangle =\left\langle |E_{z}{|}^{2}\right\rangle$ are the intensities of the in-plane (TE) and out-of-plane (TM) components of the electric field, respectively, averaged over one unit cell of the structure. The color varies from red through violet to blue, representing the average polarization state from pure TE through hybrid TE-TM to pure TM. The opacity of the color (on a white background) represents the absorptance. Figure 3(a) shows the anticrossing of the simple TE and TM WPPs (red and blue bands, respectively), resulting in hybrid TE-TM branches (violet) and a high-*Q* BIC between them. In this case, the intersection of the Bragg conditions for the TE and TM modes (red and blue dashed lines, respectively) overlaps with the LSPR, which makes this scenario similar to that of Figure 1(b). In this example, the nanoparticle size is relatively large (75 nm), which causes a significant hybridization, splitting the modes far apart in the spectrum. In contrast, Figure 3(b) and (c) show analogous results, but for metasurfaces with much smaller nanoparticles (25 nm). Due to the smaller size, the splitting is significantly smaller. In addition, the LSPR of the nanoparticles is shifted to a shorter wavelength. In Figure 3(b), the values of Λ_*x*_ and Λ_*y*_ are adjusted such that the intersection between the TE and TM Bragg conditions matches the LSPR, as in Figure 3(a). In contrast, in Figure 3(c), the TE and TM Bragg conditions are detuned far from the LSPR, and the anticrossing picture resembles that in Figure 1(c), apparently involving only two interacting modes. However, in all the presented examples, the BIC emerges near the TE-TM intersection point, resulting from the actual three-mode coupling mechanism described by the Hamiltonian in Eq. (2).

**Figure 4:**
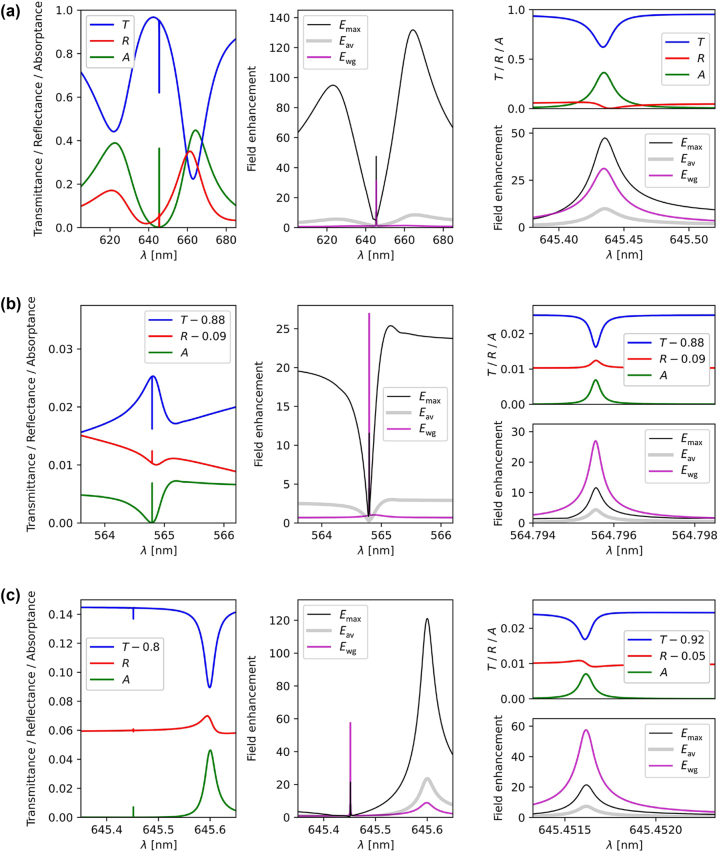
Spectral dependence of the transmittance (*T*), reflectance (*R*), absorptance (*A*) (left column), and the values of the local field enhancement at the surface of the nanocubes (maximum *E*_max_ and average *E*_av_) and inside the waveguide (*E*_wg_) (middle column). The plots in (a), (b), and (c) reveal the quasi-BICs at Λ_*x*_ = 352.5 nm, 299.75 nm, and 352.47 nm in Figure 3(a)–(c), respectively. Graphs in the right column show the magnifications around the quasi-BICs of the graphs in the left and middle column. The values of transmittance, reflectance, absorptance and field enhancement were obtained using COMSOL Multiphysics.

**Figure 5:**
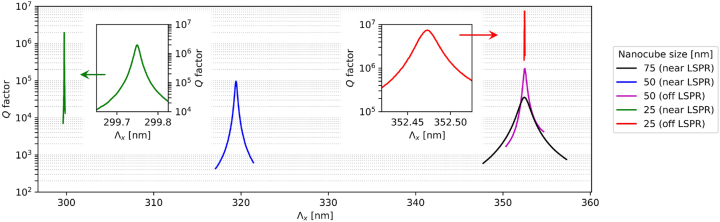
*Q* factors of the hybrid quasi-BICs as functions of Λ_*x*_ in metasurfaces of various parameters (see legend). The black, green, and red curves correspond to the metasurfaces in Figures 3(a)–(c) and 4(a)–(c), respectively. Λ_*y*_ is set to 340 nm for the black, magenta, and red curves, 310 nm for the blue curve, and 292 nm for the green curve. The curves for 25 nm nanocubes (green and red lines) are magnified in the insets. All values were extracted from the spectral FWHM of the absorptance peaks calculated using COMSOL Multiphysics.

Figure 4 shows the spectral dependence of transmittance (*T*), reflectance (*R*), absorptance (*A*), and field enhancement (maximum *E*_max_ and average *E*_av_ at the nanoparticle surface, as well as average *E*_wg_ in the waveguide). The plots presented in Figure 4(a)–(c) are obtained assuming the same metasurface geometries as in Figure 3(a)–(c), respectively, but each for a fixed value of Λ_*x*_. In other words, the plots in Figure 4 are vertical cross-cuts of the two-dimensional plots in Figure 3. In each case, the value of Λ_*x*_ is selected such that the spectra in Figure 4 reveal the narrowest BIC peaks. Some of the curves in Figure 4(b) and (c) are vertically shifted (*T* − 0.88, *R* − 0.09, etc.) to make the BIC peaks better visible. We determine the *Q* factors of these BICs from the full width at half maximum (FWHM) of their absorptance peaks. The obtained dependence of the *Q* factor versus Λ_*x*_ is presented in Figure 5 for various nanoparticle sizes (25, 50, and 75 nm).

In the case of 75-nm nanoparticles (see Figures 3(a) and 4(a), and the black curve in Figure 5), the highest value of *Q* is found to be about 3.5 × 10^4^. On the other hand, for the smaller 25-nm nanoparticles (see Figures 3(b), 3(c), 4(b), and 4(c), and the green and red curves in Figure 5), the *Q* factor can exceed 10^6^, reaching 2.0 × 10^6^ in the resonant case and 7.4 × 10^6^ in the off-resonant case. This dependence of the maximum *Q* factor on the nanoparticle size results from the fact that, in our examples, the finite-sized nanoparticles slightly modify the two interacting lossless modes. Ideally, if the nanoparticles were infinitely small, the electric fields of these modes would interfere to find a configuration with a zero intensity at the positions of the nanoparticles. In such an ideal case, the resulting BIC would be perfectly lossless and at the same time it would remain a hybrid eigenmode coupled to the LSPR of the nanoparticles, as predicted by the model in Eq. (2). To achieve a perfectly lossless hybrid BIC with finite-sized nanoparticles, the electric field of the two lossless modes would have to be zero in a region of a finite volume. This is impossible, as it would require the field to be zero everywhere according to electromagnetic theory. As a result, the *Q* factor remains finite as long as the nanoparticles do not behave as point dipoles. Furthermore, the efficiency of the loss cancellation in a real system depends on the validity of the approximations used to derive the Hamiltonian ***H*** in Eq. (2) from Eq. (1). For example, the interaction picture can be affected by the contribution of other modes (apart from the three modes considered here) or by the presence of unwanted coupling between the two lossless modes (other than the coupling mediated by the lossy mode).

Imperfect implementation of the model by the considered metasurfaces with finite-sized nanoparticles turns the proposed BICs into *quasi-BICs*. These resonances are no longer fully decoupled from the free-space radiation. Hence, they can be excited by the incident light and show narrow spectral features, as can be seen in Figure 4. By making the nanoparticles smaller, the metasurface gets closer to the ideal case described by the model. As a result, the *Q* factor of the quasi-BIC becomes higher (see Figure 5) and the excitation by the incident light becomes less efficient. The latter leads to a decreased amplitude of the quasi-BIC peaks in the transmittance, reflectance, and absorptance spectra (see Figure 4). However, the field enhancement spectra in Figure 4 clearly show that, apart from ultrahigh *Q* factors, the quasi-BICs can produce a relatively high local field enhancement, both at the nanoparticle surface and in the waveguide, despite being relatively dark. This is because the high-*Q* resonance associated with the quasi-BIC allows the light to be efficiently trapped in the metasurface. Consequently, its amplitude builds up via constructive interference in all locations, including the lossy nanoparticles that it tries to avoid. The interaction of light with the nanoparticles may in this case be strongly enhanced within a narrow resonance band [56–59]. These properties make the presented quasi-BICs very promising for applications in optical sensing [104, 105] and strong light–matter coupling [49]. We emphasize that, in practice, ultrahigh *Q* factors can be deteriorated by fabrication imperfections, finite illumination area (i.e., finite width of the angular spectrum of the incident beam) [106], and finite lateral extent of the metasurface (side leakage) [13, 44, 107]. However, the proposed BICs are rather insensitive to the shape of the nanoparticles, which makes them robust and experimentally feasible compared to typical symmetry-enabled BICs [108]. Further improvement of the proposed metasurface implementation could involve, e.g., using silver instead of gold as the nanoparticle material, or tuning the resonances to longer wavelengths, at which plasmonic metals behave more like perfect conductors. This could possibly enable one to further reduce the size while maintaining a high scattering cross section of the nanoparticles necessary for efficient coupling between the LSPR and the guided modes.

## 4 Summary and conclusions

To summarize, we have demonstrated the possibility of simultaneous suppression of the radiation and absorption losses in a plasmonic metasurface, creating BICs with *Q* factors as high as 10^6^ – 10^7^, that can be further increased by optimizing the structure (e.g., replacing gold with silver). The presented mechanism of loss cancellation can be implemented in any system of two initially uncoupled and lossless oscillators interacting with a third oscillator that can have arbitrary losses. In this case, the condition for achieving an absorption-free BIC corresponds to the exact matching between the resonance frequencies of the two lossless oscillators. In the example system – a slab waveguide and an array of plasmonic nanoparticles – the TE and TM guided modes played the role of the two lossless oscillators, while the LSPR of the plasmonic nanoparticles served as the third lossy oscillator. The presented mechanism is not limited to the above specific example and can be realized in other physical systems within and beyond optics.

In the context of optical BICs, simultaneous elimination of both the radiation and absorption losses could open up many possibilities. In plasmonics, for example, it could enable high-*Q* resonances with strong local field enhancement, improving the performance of plasmonic systems [45, 65, 109]. On the other hand, high-index dielectrics (e.g., Si, Ge, and GaAs), which are strongly absorbing in the visible spectral range, could be used to create high-*Q* Mie-resonant photonic structures [110]. This can be very useful in all applications relying on resonant enhancement of light–matter interaction, including ultrasensitive spectroscopic devices, nonlinear optical modulators and frequency converters, and light emitters with tailored characteristics [111–113].
